# UWB Antenna with Enhanced Directivity for Applications in Microwave Medical Imaging

**DOI:** 10.3390/s24041315

**Published:** 2024-02-18

**Authors:** Dawar Awan, Shahid Bashir, Shahid Khan, Samir Salem Al-Bawri, Mariana Dalarsson

**Affiliations:** 1 Department of Electrical Technology, University of Technology Nowshera, Nowshera 24170, Pakistan; dawar@uotnowshera.edu.pk; 2 Department of Electrical Engineering, University of Engineering and Technology Peshawar, Peshawar 25120, Pakistan; shahid.bashir@uetpeshawar.edu.pk; 3 Department of Electrical and Computer Engineering, COMSATS University Islamabad, Abbottabad Campus, Abbottabad 22060, Pakistan; shahid@cuiatd.edu.pk; 4 Space Science Center, Institute of Climate Change, Universiti Kebangsaan Malaysia (UKM), Bangi 43600, Malaysia; samir@ukm.edu.my; 5 School of Electrical Engineering and Computer Science, KTH Royal Institute of Technology, SE 100-44 Stockholm, Sweden

**Keywords:** directional antenna, microwave medical imaging (MMI), tumor sensing, ultra-wideband (UWB) antenna

## Abstract

Microwave medical imaging (MMI) is experiencing a surge in research interest, with antenna performance emerging as a key area for improvement. This work addresses this need by enhancing the directivity of a compact UWB antenna using a Yagi-Uda-inspired reflector antenna. The proposed reflector-loaded antenna (RLA) exhibited significant gain and directivity improvements compared to a non-directional reference antenna. When analyzed for MMI applications, the RLA showed a maximum increase of 4 dBi in the realized gain and of 14.26 dB in the transmitted field strength within a human breast model. Moreover, it preserved the shape of time-domain input signals with a high correlation factor of 94.86%. To further validate our approach, another non-directional antenna with proven head imaging capabilities was modified with a reflector, achieving similar directivity enhancements. The combined results demonstrate the feasibility of RLAs for improved performance in MMI systems.

## 1. Introduction

Microwave medical imaging (MMI) has emerged as a promising alternative to conventional medical imaging due to its lower cost, safety, non-ionizing radiation, and compact size [1,2,3]. MMI systems have the potential for immediate diagnosis in ambulances, and they can save lives by preventing delays [3].

MMI devices typically use antenna arrays to receive reflected and scattered signals from the target object. These signals are then processed to estimate the object’s dielectric properties, a technique known as microwave tomography (MT) [4,5,6,7,8,9,10]. In MT systems, signals are processed in the frequency domain, and antennas are arranged equidistantly around the imaging area [5,6,7,8,9,10]. Specialized algorithms estimate the object’s dielectric properties based on the reflected signals.

Alternatively, radar-based MMI approaches aim to locate objects based on reflected signals. This (confocal) imaging method offers faster processing and lower computational complexity by processing signals in the time domain. Examples of such radar-based systems for biomedical imaging were presented in [11,12,13].

Another exciting application of MMI is noninvasive temperature monitoring during hyperthermia treatment, where the tumor temperature is intentionally raised to around 44 °C for at least one hour [4]. MMI can be used to monitor the tumor’s dielectric properties, with changes in temperature being reflected by changes in these properties.

MMI also offers the possibility of 3D visualization of a region of interest (ROI) through various reconstruction algorithms. Such three-dimensional reconstructions were reported in [5,6].

Recently, researchers have explored combining MT and radar-based approaches to leverage the strengths of both. MT provides the dielectric profile of the object under consideration, offering the possibility to detect the presence of tumors or cancerous cells [14,15]. The radar-based approach can then be applied only to suspected areas, significantly speeding up MMI reconstruction [14,15]. These advancements necessitate new requirements for antenna elements.

A wider operating bandwidth, greater signal penetration capabilities, a good fidelity factor or correlation factor in the time domain, and higher values of gain and directivity are generally the desirable characteristics of an antenna for its use in MMI. Among several UWB antennas reported for MMI applications, popular choices include Vivaldi planar antennas [15,16], double-ridged horn antennas [17,18], planar monopoles [6,19,20], and bowtie dipoles [21,22]. While the above-mentioned antennas have proven capabilities for their use in MMI, there is always room for improvement in antenna performance.

This work aims to improve the performance of antennas for microwave medical imaging (MMI) systems by focusing on their radiating properties. Specifically, it seeks to enhance their directivity for better imaging results. Recent studies have explored various methods of achieving this goal. Reference [23] utilized a four-layer lens and modified antenna to generate focused near-field radiation, achieving a 4.9 dB increase in penetrated signal strength. While commendable, this work demonstrated a gain improvement of 14.26 dB. Another approach that was detailed in [24] employed a balanced-to-unbalanced circuit within a conventional UWB bowtie antenna, successfully reducing backward radiation. However, this technique applied only to the bowtie design. This work proposed a more general solution for mitigating backward radiation across various MMI antennas. Reference [25] presented an eagle-shaped UWB patch antenna for MMI, emphasizing its novel shape for directional radiation. In comparison, this work proposes a simpler and more effective technique for achieving a significantly more directional radiation pattern. Building upon concept of etching slots and adding parasitic patches in [26], this work presents a technique that is applicable to various printed UWB antennas. It offers a more notable gain improvement (over 4 dB) compared to the 1.5 dB increase reported in [26]. While previous research, such as that of [27,28,29], explored signal processing and algorithms for MMI systems, this work focuses solely on antenna performance and on specifically enhancing directivity for improved MMI.

This study investigates the use of reflection and electromagnetic (EM) wave focusing to boost antenna gain. This approach is widely used in directional antennas across various applications, including satellite communication, cellular networks, radar systems, and even medical technologies.

Our core concept involves directing EM radiation in a specific direction to achieve high gain and, consequently, high directivity. This aligns with the established need for directional ultra-wideband (UWB) antennas in microwave medical imaging (MMI) systems, where antennas with strong, focused radiation have proven superior to non-directional or low-gain alternatives.

A compact slotted patch antenna [30] with good MMI properties was chosen as the reference antenna. Drawing inspiration from the highly directional Yagi-Uda antenna, which utilizes a large reflector and parasitic directors to focus radiation, this work proposes loading the reference antenna with a metallic reflector.

The resulting reflector-loaded antenna (RLA) exhibited encouraging results, including increased directivity and directional radiation patterns throughout the operating band. Its suitability for MMI was analyzed based on the field strength inside human tissues, time-domain properties, and frequency-domain performance. The analysis demonstrated the RLA’s superior performance compared to that of the reference antenna in MMI applications. 

A comparison of the performance of directional and omnidirectional antennas for wide band head imaging is also presented in [31]. However, to the best of our knowledge, no work has presented a generalized technique for enhancing the directivity of antennas for use in MMI systems. This work presents a simple and novel technique that can convert a wide class of non-directional antennas into directional ones. The novelty of this work also rests on the fact that the application of the proposed technique is not limited to a single antenna, but it is applicable to a wide range of antennas. It is exhibited with simulated and experimental evidence that by loading a printed antenna with a metallic reflector, the antenna’s directivity can be improved.

In this work, a generalized technique is presented to enhance the gain and directivity of a wide range of printed UWB antennas based on the design formulas of the Yagi-Uda antenna. The size and position of the reflector for a printed UWB antenna were related to the design formulas of the Yagi-Uda antenna; this is a novel approach, and such a correspondence has not been explicitly presented in any previous work performed on antenna design for MMI systems.

This work presents a very simple technique that significantly increased the gain of a printed UWB antenna by 4 dBs (further increasable) without deteriorating the antenna’s operating bandwidth, efficiency, and time-domain properties. Most of the available literature that focused on increasing an antenna’s gain or directivity presented complicated antenna designs based on techniques that are not applicable to any other antennas. This work presents a simple general technique and demonstrates its applicability to two different antennas. A thorough analysis of the performance of the proposed directional antenna for applications in MMI was performed based on the field distribution inside the object to be imaged, the time-domain performance of the antenna, and the frequency-domain performance of the proposed antenna. The proposed antenna caused EM waves to penetrate inside a breast model with an increase in the signal strength of 14.26 dB. The frequency-domain analysis proved that the proposed antenna was much more sensitive to the detection of the existence of a tumor than the reference antenna was. The proposed antenna offered a time-domain pulse fidelity factor of 94.86%.

There is very little work in the literature that focused on directional antennas for MMI purposes. This work proposes a novel reflector-loaded antenna with a highly directional radiation pattern for applications in MMI systems. When compared in terms of performance parameters, the proposed antenna outperformed other designs proposed for MMI systems.

In the following sections, a detailed discussion of antenna design, the proposed technique, and the feasibility of the application of the proposed antenna in MMI systems is presented. To analyze the antenna performance in MMI systems, a human breast is simulated, and an analysis of the field distribution is conducted, followed by a rigorous analysis of the antenna’s time-domain performance in the E-plane and H-plane. A frequency-domain analysis was also performed to highlight the superior performance of the proposed antenna for MMI applications. Before presenting the conclusion of this work, the analysis was repeated for another reference antenna in order to establish the generalizability of the proposed technique. Later, a performance comparison of the proposed antenna with those in other similar works is described.

## 2. Antenna Design

### 2.1. Reference Antenna

**Figure 1:**
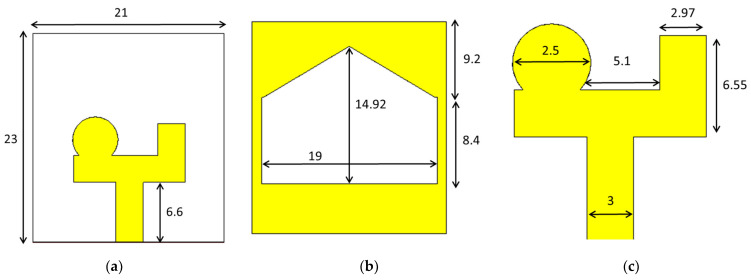
(**a**) Front view, (**b**) back view, and (**c**) radiating patch of the reference antenna (all dimensions are in mm).

**Figure 2:**
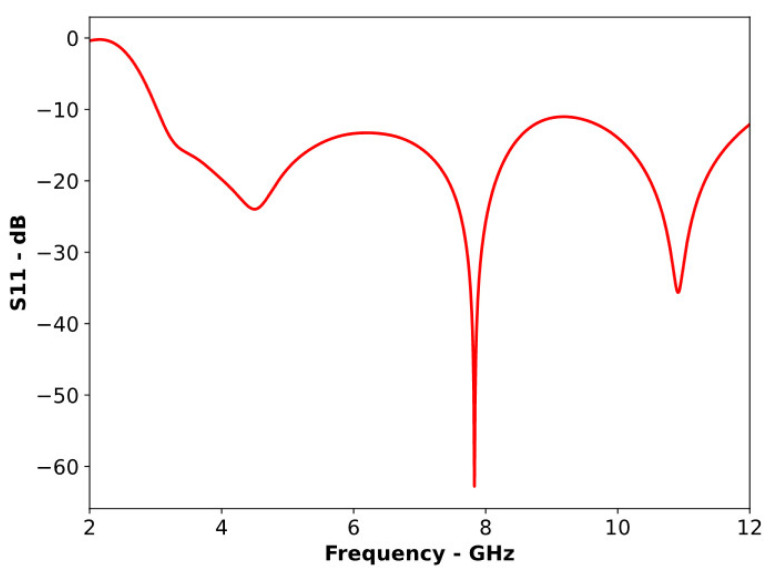
S11 plot of the reference antenna.

**Figure 3:**
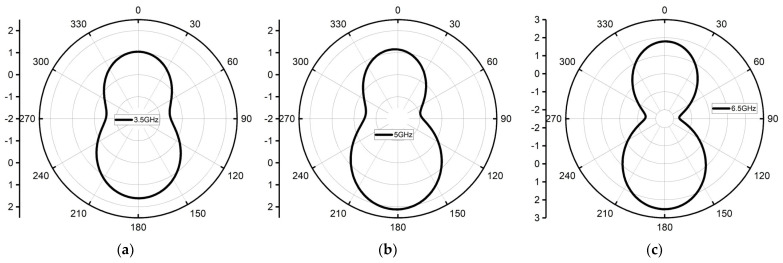

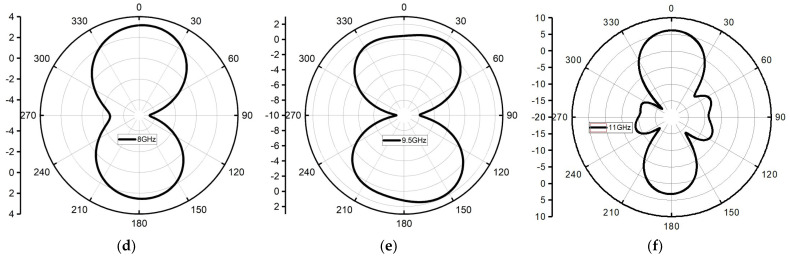
Radiation pattern of the reference antenna for Φ=0° and varying values of θ for frequency = (**a**) 3.5, (**b**) 5, (**c**) 6.5, (**d**) 8, (**e**) 9.5, and (**f**) 11 GHz.

**Figure 4:**
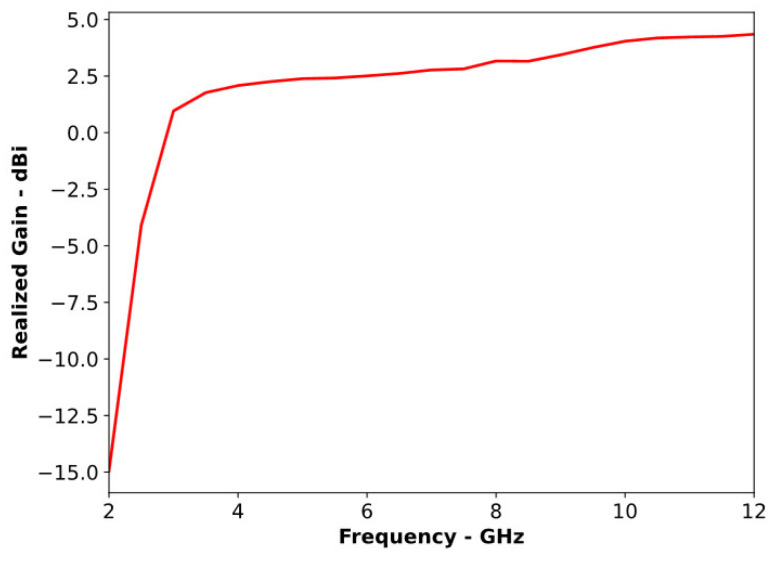
Gain of the reference antenna.

A compact slotted patch antenna, shown in Figure 1, was simulated in CST MWS. The antenna had overall dimensions of 21 × 23 × 1.6 mm and an operating bandwidth of 3.02 to 11.36 GHz. As shown in Figure 2, the antenna exhibited good matching properties across its working band. The antenna’s radiation pattern, presented in Figure 3, clearly illustrates its non-directional radiation properties. This consistent non-directional radiation made the antenna a good candidate for conversion into a directional version for a comparison of its performance in MMI applications. Figure 4 shows that the antenna exhibited an almost uniform gain across most of its operating bandwidth.

The initial length and width of the radiator are based on the following design equations [32]:

(1) $$W_{p}=\frac{c}{2f_{r}}\sqrt{\frac{2}{\varepsilon_{r}+1}}$$

(2) $$\mu_{reff}=\frac{\mu_{r}+1}{2}+\frac{\mu_{r}-1}{2}{\left[1+12\frac{h}{W}\right]}^{-\frac{1}{2}}$$

(3) $$L_{p}=\frac{c}{{2f}_{r}\sqrt{\mu_{reff}}}$$

In the above equations, $f_{r},\;c,\;\varepsilon_{r},\;h$, and $\varepsilon_{reff}$ represent the resonant frequency, speed of light, material permittivity, substrate height, and effective dielectric constant, respectively.

### 2.2. Proposed Design

**Figure 5:**
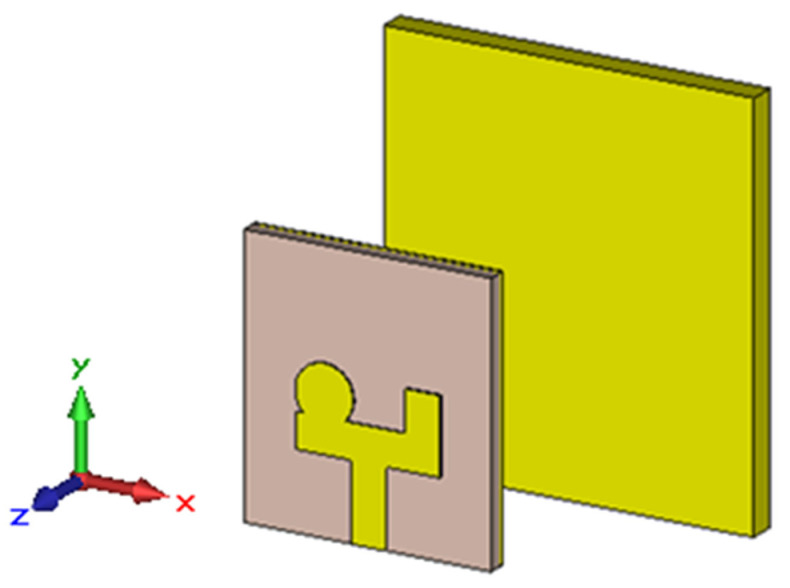
The configuration of the reflector-loaded antenna.

**Figure 6:**
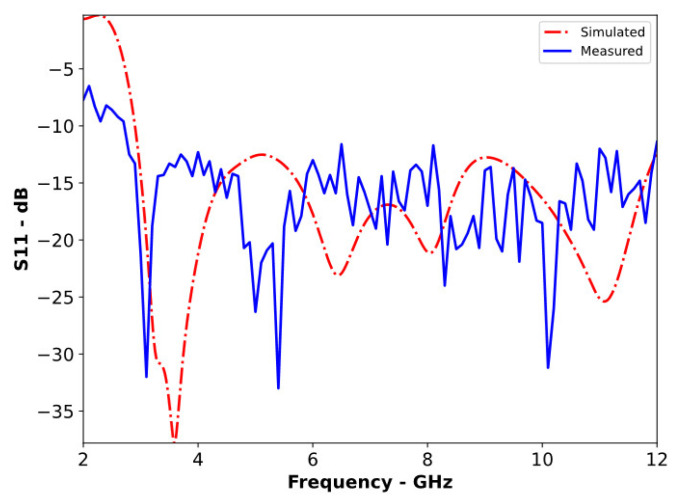
Simulated and measured S11 of the RLA.

To enhance the directivity, the reference antenna was loaded with a reflector, as shown in Figure 5. Following the Yagi-Uda antenna design principles, the reflector was positioned 25 mm (0.25λ at the lowest operating frequency) from the radiating element. This arrangement ensured acceptable matching characteristics for the RLA across its entire operating bandwidth, as shown in Figure 6.

The design formulas suggested a reflector size slightly larger than 0.5λ when the driven element was between 0.45λ and 0.49λ [33]. As shown in Figure 1, the reference antenna’s horizontal dimension was 21 mm (about half of 0.45λ). Following this principle, the reflector’s horizontal dimension was set to 29.4 mm (approximately half of 0.6λ), ensuring conformity with the Yagi-Uda design principles.

**Figure 7:**
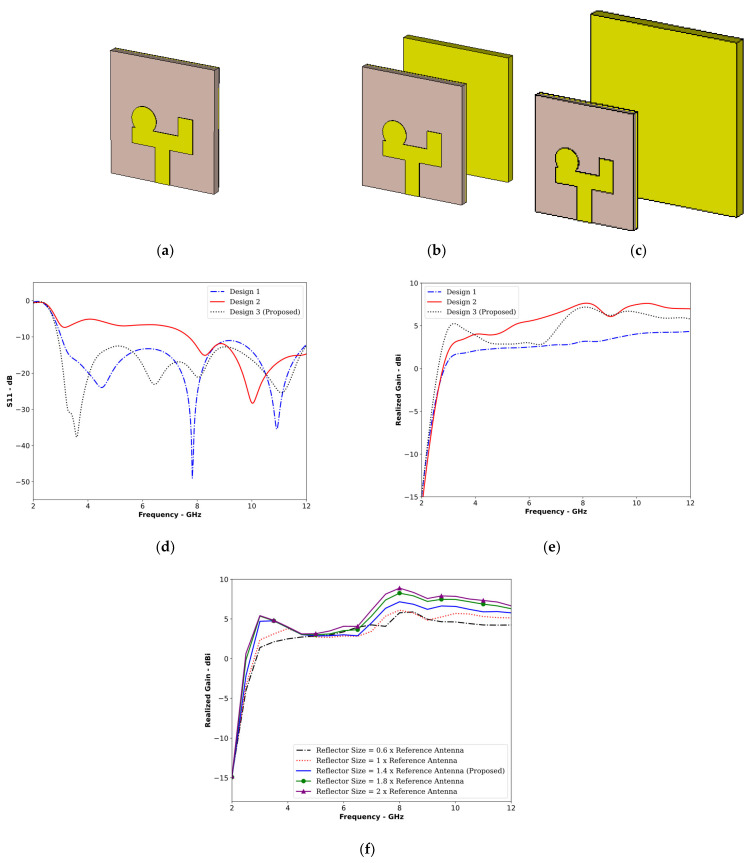
Design evolution of the proposed antenna. (**a**) Design 1, (**b**) Design 2, (**c**) Design 3 (**d**) S11 plots, (**e**) plots of the realized gain of the three designs, and (**f**) the effect of reflector size on antenna gain.

Figure 7 illustrates the evolution of the proposed antenna design, which was broken down into three key stages: Design 1, Design 2, and the final proposed Design 3. Drawing inspiration from the Yagi-Uda antenna’s high directivity, this work focused on optimizing the reflector’s design and placement for enhanced gain and directivity. Design 1 serves as the reference antenna. Design 2 introduces a rectangular reflector, which was chosen for its simplicity. However, placing the reflector at a random distance in Design 2 led to significant impedance mismatch and reduced operating bandwidth, as shown in Figure 7d. Leveraging design formulas from the Yagi-Uda antenna, careful calculations identified the optimal position and size for the reflector, resulting in the proposed Design 3. This final design achieved the same bandwidth as that of the reference antenna while offering a significant gain improvement (detailed in Figure 7e). Further analysis of the directivity and radiation patterns will be presented in subsequent sections. Figure 7f presents a parametric analysis of the effect of varying reflector size on the realized gain of the antenna. It can be seen in Figure 7f that by increasing the reflector size, higher values of gain can be achieved. This also implies that by increasing the reflector size, the directivity of the antenna can also be further enhanced. However, a reflector size of 1.4 times that of the reference antenna was used to ensure conformity with the Yagi-Uda design formula, as discussed above.

**Figure 8:**
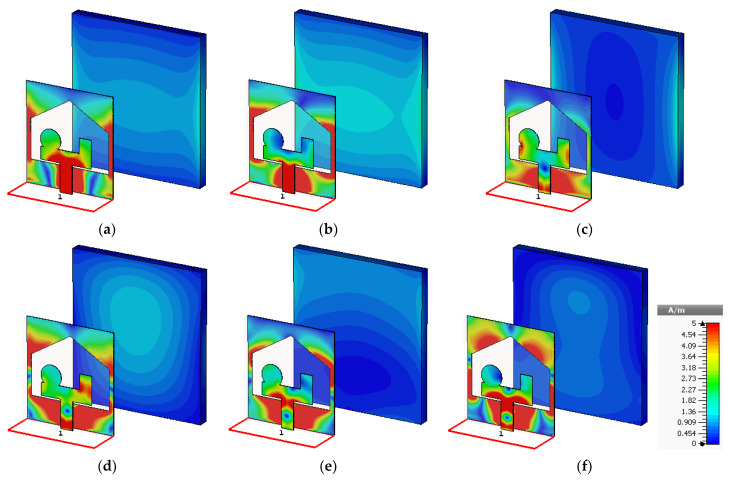
Surface current distribution of the proposed RLA at (**a**) 3.5, (**b**) 5, (**c**) 6.5, (**d**) 8, (**e**) 9.5, and (**f**) 11 GHz.

Figure 8 shows the surface current distribution at different frequencies within the antenna’s operating bandwidth. Initially, the current was primarily concentrated on and around the feeding line. However, as the frequency increased, the current density on the feeding line decreased and started to spread more evenly across the ground plane beneath it.

**Figure 9:**
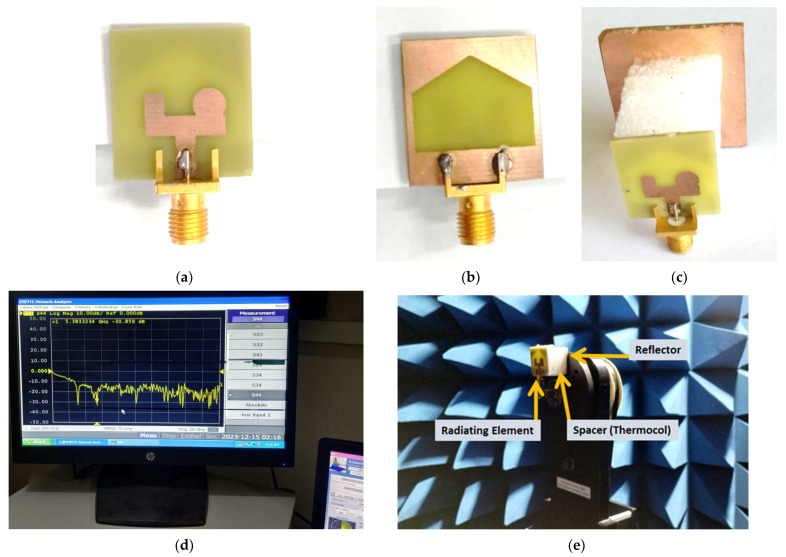
(**a**) Front and (**b**) back view of the reference antenna; (**c**) the proposed RLA; (**d**) experimental setup with VNA; (**e**) radiation pattern measurement inside an anechoic chamber.

**Figure 10:**
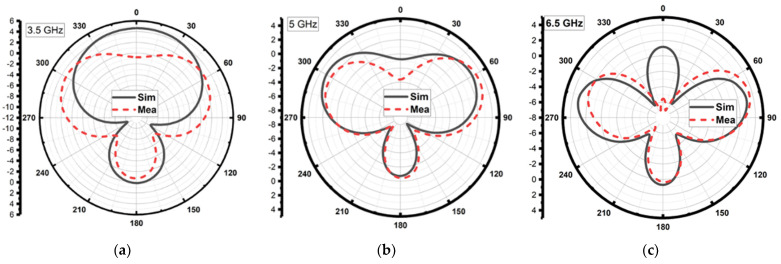

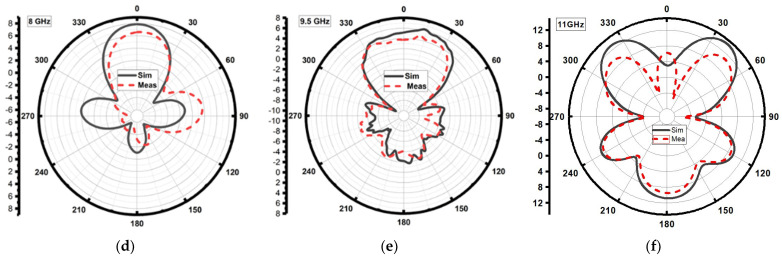
Radiation pattern of the proposed RLA for Φ=0° and varying values of θ for frequency = (**a**) 3.5, (**b**) 5, (**c**) 6.5, (**d**) 8, (**e**) 9.5, and (**f**) 11 GHz.

**Figure 11:**
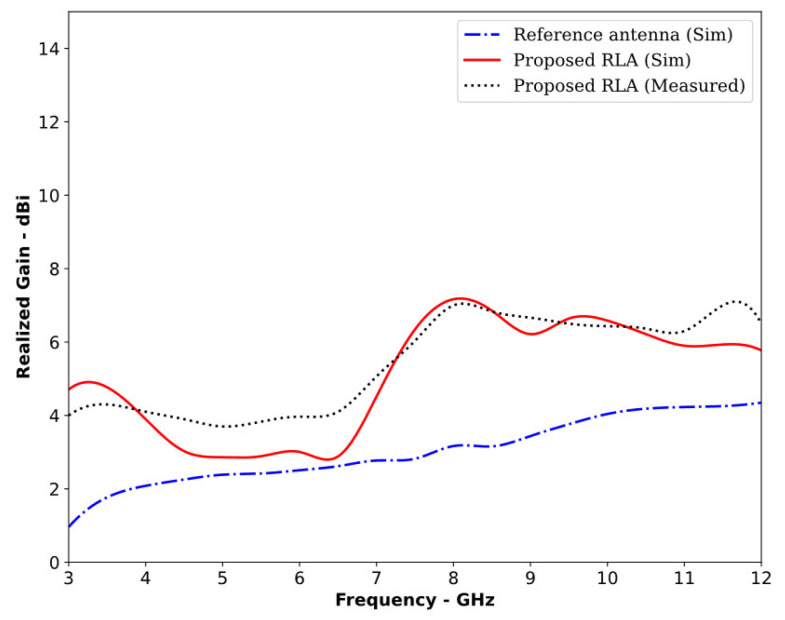
Realized gain (simulated) of the reference antenna and proposed RLA and measured gain of the proposed RLA.

**Figure 12:**
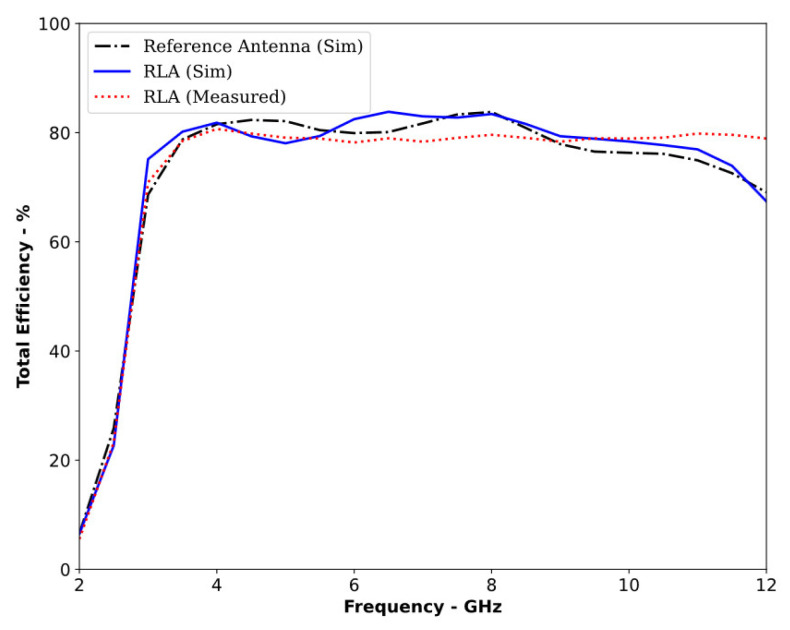
Total efficiency of the reference antenna and proposed RLA (simulated and measured).

The proposed RLA was fabricated on a cost-effective FR-4 substrate with a dielectric constant of 4.4 and a loss tangent of 0.02. Laser etching was used to precisely define the top and bottom layers of the antenna design. A simple rectangular copper sheet served as the reflector, while a 50 ohm SMA connector, which was carefully soldered to ensure proper matching, provided a connection to the VNA. To measure the radiation pattern across its operating bandwidth, the antenna was tested in an anechoic chamber through a series of trials. Figure 9 depicts both the antenna design and the experimental setup. Figure 10 confirms that the RLA functioned as a directional antenna throughout the operating bandwidth, as expected. Loading the reference antenna with the reflector significantly altered its radiation properties. The RLA exhibited a notable increase in gain across the entire band, reaching a maximum of 7.3 dB at 10.5 GHz and achieving a peak gain of 30 dB at 11 GHz, as shown in Figure 11. Fair conformity can be observed in the measured and simulated results in Figure 10 and Figure 11. Figure 12 depicts the total efficiency of the proposed RLA (simulated and measured) and the reference antenna; it can be seen that the addition of the reflector did not deteriorate the total efficiency of the antenna; in fact, at some frequency points, the efficiency was improved due to the presence of a reflector.

The proposed RLA achieved a directional radiation pattern with a maximum increase of 4 dBi in the realized gain compared to the reference antenna, leading to improved signal focusing and reduced interference. This is further evident in Table 1, which summarizes the RLA’s enhanced performance across various parameters.

## 3. Application of the Proposed Antenna in Microwave Medical Imaging Systems

For MMI applications—in particular, tumor detection and localization across various regions of the body—the desirable antenna characteristics include a wide operating bandwidth, high signal penetration capabilities, good time-domain fidelity or correlation factor, high gain at lower frequencies, and high directivity. High values for these parameters generally lead to better image quality and resolution. To evaluate the performance of the proposed RLA, we conducted simulations and compared the results against these desired characteristics.

### 3.1. Field Analysis

This section analyzes the performance of the reference antenna and the proposed RLA for their application in MMI. A simulation setup was used to model a human breast (ε=38) with a tumor (ε=67) and to compare the E-field distribution generated by each antenna.

**Figure 13:**
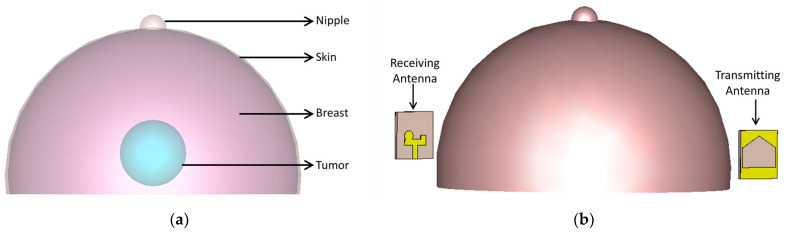
(**a**) Breast model and (**b**) simulation setup of the breast model placed between two reference antennas for imaging purposes.

Figure 13 depicts the simulation setup. A human breast model containing a tumor was positioned between two antennas. Antenna 1 acted as the transmitter, while Antenna 2 received the signals.

**Figure 14:**
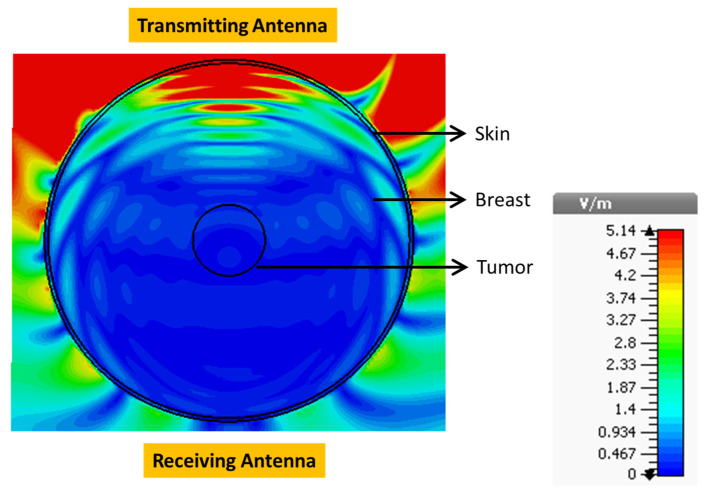
E-field distribution in the breast (top view) when placed between two reference antennas.

The E-field distribution within the model at 4.6 GHz is shown in Figure 14. The maximum field strength inside the tumor was 0.3618 V/m, and this was located at the position of the yellow cursor.

**Figure 15:**
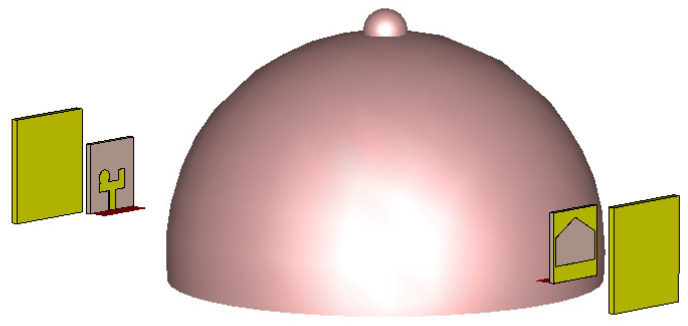
Simulation setup of a human breast model placed between two proposed RLAs.

**Figure 16:**
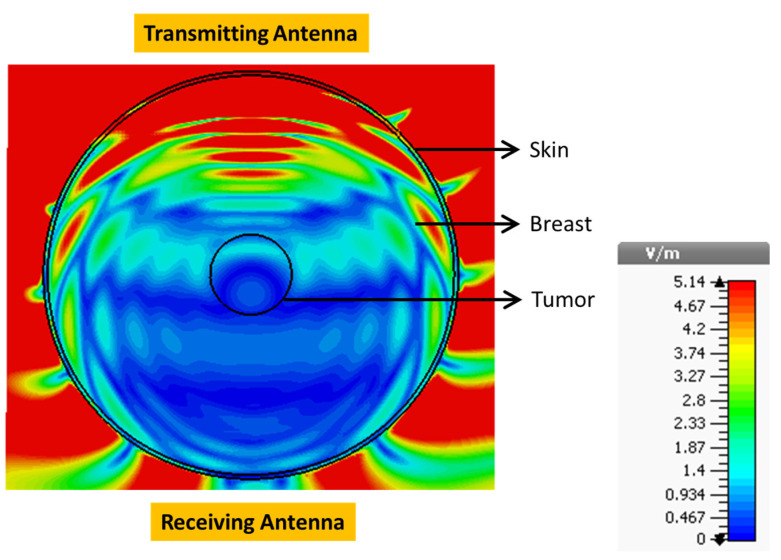
E-field distribution in the breast when placed between two proposed RLAs.

Repeating the simulation with RLAs instead of the reference antennas (Figure 15) revealed a significantly higher E-field distribution inside the breast (Figure 16). The maximum field strength inside the tumor reached 1.56 V/m at the same location. This represented a 12.73 dB increase in field strength achieved by the RLA.

This remarkable increase in field strength was attributed to the RLA’s higher directivity, leading to improved tumor sensing and imaging results. Table 2 further confirms the RLA’s superior performance. The RLA consistently generated significantly higher E-field strength inside the tumor compared to the reference antenna across the entire frequency spectrum. Notably, the RLA exhibited a minimum increase of 11.68 dB at 5 GHz and a maximum increase of 14.26 dB at 3.5 GHz, demonstrating its superior signal penetration capabilities within human tissues.

Table 3 compares the maximum E-field values inside the tumor when both antennas were positioned sideways to the breast model. The RLA consistently achieved higher field values, with a maximum increase of 5.40 dB at 6.5 GHz. This demonstrated the RLA’s effectiveness in both orientations, facing the breast model and sideways with respect to it.

The RLA exhibited a significantly higher field penetration capability than that of the reference antenna, leading to improved tumor detection and imaging resolution. Its effectiveness in both orientations further enhanced its suitability for MMI applications.

### 3.2. Time-Domain Analysis

**Figure 17:**
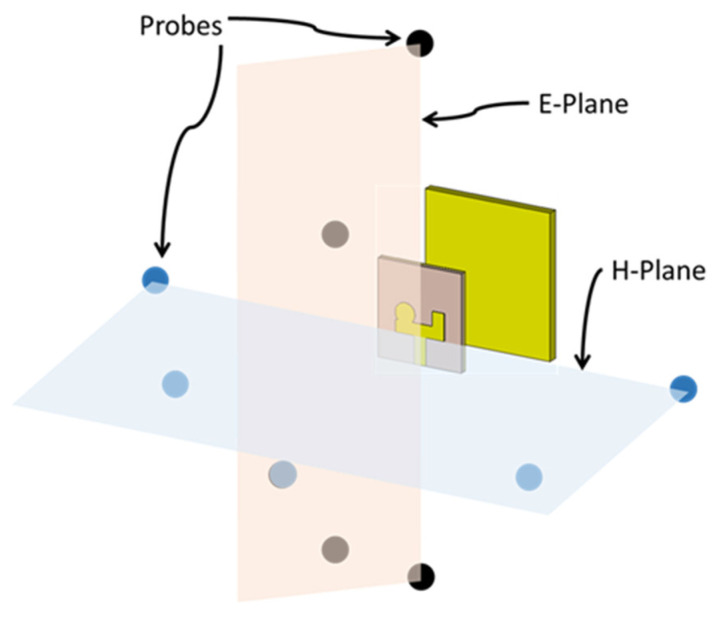
Configuration of the virtual probes placed in the E-plane and H-plane.

This section examines the time-domain performance of the RLA, analyzing its suitability for MMI applications. Inspired by [34,35], a comprehensive study of the transceiver performance was conducted. Five virtual probes were positioned in the E-plane and H-plane of the antenna to capture received signals at various angles. Figure 17 shows the probe configuration.

**Figure 18:**
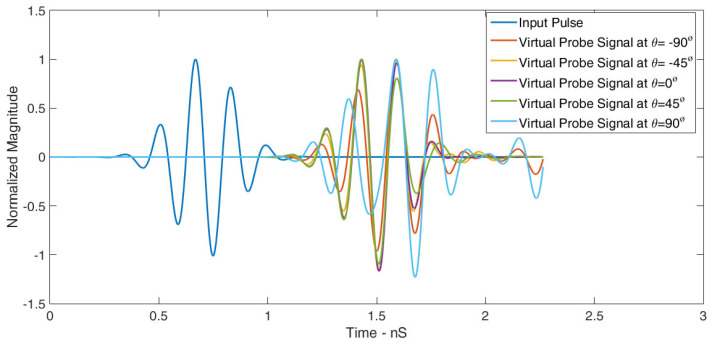
Normalized signals received by virtual probes placed in the E-plane for ∅=90° and varying values of θ.

A Gaussian pulse served as the input signal at the transmitting antenna port, and the signals were collected by the virtual probes. Figure 18 illustrates the normalized input and received signals in the E-plane for different angles. The well-preserved signal shapes demonstrate the RLA’s excellent signal fidelity.

**Figure 19:**
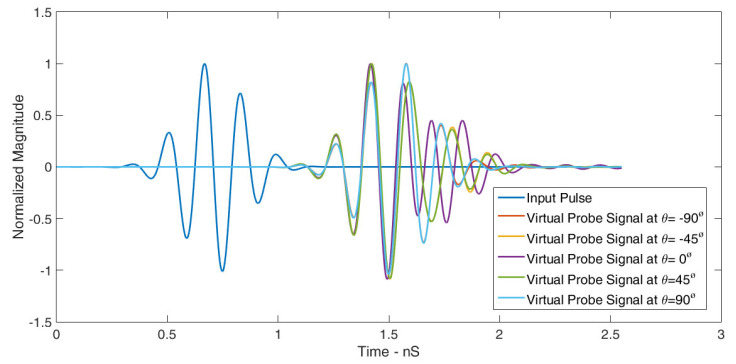
Normalized signals received by virtual probes placed in the H-plane for ∅=0° and varying values of θ.

Table 4 summarizes the correlation factors for this scenario. The RLA exhibited exceptional signal matching in the E-plane, with a maximum correlation factor of 98.79% and a minimum of 80%. Similar findings can be observed in Figure 19, where five probes were placed in the H-plane at equal angular distances. Table 5 presents the correlation factors for these probes, revealing a maximum of 98.82% and a minimum of 84%. These results confirm the RLA’s outstanding transceiver performance in both planes. The following equation is used to calculate the correlation factor.

(4) $$C.F={max}_{\tau }\left|\frac{\int_{-\infty }^{\infty }s\left(t\right)r(t-\tau )}{\sqrt{\int_{-\infty }^{\infty }{s(t)}^{2}dt.\int_{-\infty }^{\infty }{r(t)}^{2}dt}}\right|$$

where $s\left(t\right)$ is the time-domain input signal, $r\left(t\right)$ is the time-domain signal received at the port of the receiving antenna, and τ is the delay between s(t) and $r\left(t\right),$ which is varied in order to achieve the maximum value of the correlation factor (C.F) in Equation (4).

**Figure 20:**
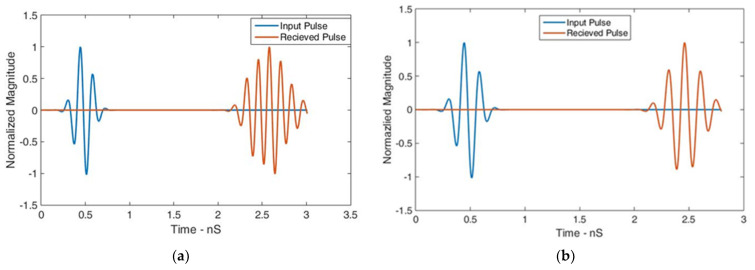
Normalized transmitted and received pulses in the (**a**) face-to-face and (**b**) side-by-side configurations with a separation of 500 mm between the RLAs.

In another simulation setup, the RLAs were placed face to face and side by side. Figure 20 shows the received pulse shapes, indicating satisfactory dispersive behavior. The correlation factors between the input and received pulses were calculated as 86.33% and 94.86% for the face-to-face and side-by-side configurations, respectively.

The analysis of the time-domain properties demonstrated the RLA’s suitability for MMI applications. For comparison, the reference antenna’s correlation factors were determined to be 94.54% and 94.59% for the face-to-face and side-by-side configurations, respectively. The decreased value of the correlation factor in the face-to-face configuration of the proposed antenna was due to the ground bounce losses taking place due to the placement of the reflector.

The above discussion clearly compares the properties of the reference antenna and the proposed RLA for MMI applications. The RLA exhibited superior time-domain properties and increased directivity, resulting in improved signal penetration within the human body. This translated into better sensing and imaging results. Loading the reference antenna with a reflector effectively increased its directivity while maintaining favorable time-domain properties, making the RLA a more desirable candidate for MMI systems.

### 3.3. Frequency-Domain Analysis

To further strengthen our analysis, we analyzed the S21 parameters of the reference antenna and the proposed reflector-loaded antenna (RLA). S21 represents the signal received at the receiving antenna in the frequency domain. By comparing the S21 curves obtained for a healthy and cancerous breast, we can effectively detect the presence of a tumor. A larger difference between these curves indicates more precise tumor detection.

**Figure 21:**
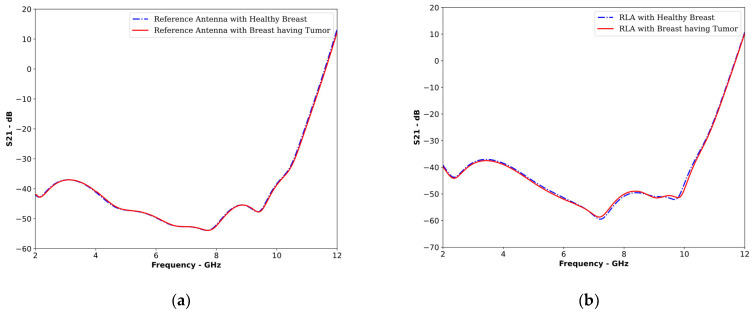
S21 plots of healthy and cancerous breast models with (**a**) reference antennas and (**b**) the proposed reflector-loaded antennas.

Figure 21 clearly demonstrates the improved tumor detection with the RLA. In Figure 21a, the S21 curves for the healthy and cancerous breast with the reference antenna are nearly indistinguishable, suggesting that there were similar received signals in the frequency domain. However, in Figure 21b, the S21 curves using the RLA are visibly different, indicating that there was a clear differentiation between the received signals for healthy and cancerous tissues.

Since the use of reference antennas for MMI applications has already been established, the significantly improved signal differentiation achieved with the RLA confirmed its superiority in tumor detection.

It is now evident that the proposed RLA can work as a better sensor for tumor detection in MMI systems as compared to the reference antenna. While analyzing both antennas across various metrics, Table 6 reveals the proposed RLA’s superior performance in enhancing the field strength within the tumor and maintaining stable signal fidelity (correlation factor) compared to the reference antenna. Notably, the RLA demonstrated a consistent difference in S21 with the presence of a tumor as compared to the reference antenna.

## 4. Generality of the Technique

**Figure 22:**
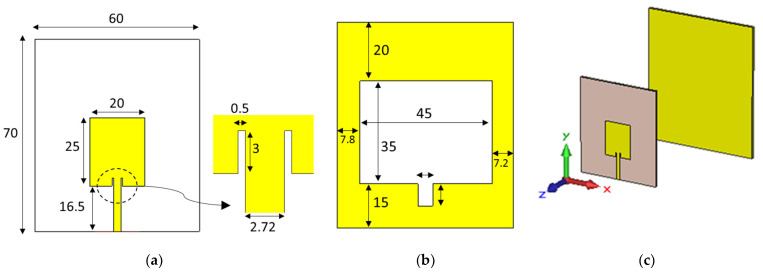
(**a**) Front and (**b**) back sides of RA2 and (**c**) the proposed RLA2 (all dimensions are in mm).

To validate the proposed reflector-loading technique’s generalizability, another antenna (RA2) reported in [36] was studied. This slotted monopole patch antenna (shown in Figure 22a,b) was identified as a promising candidate for microwave head imaging, since it was reported to have an omnidirectional radiation pattern. This characteristic made it ideal for conversion into a directional antenna.

**Figure 23:**
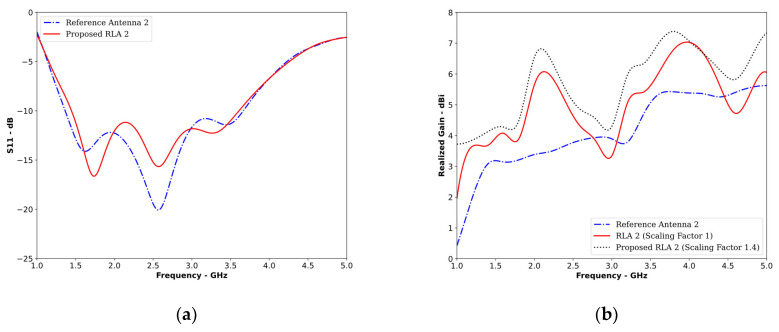
(**a**) S11 and (**b**) realized gain of RA2 and RLA2.

Adding a metal reflector, as shown in Figure 22c, significantly increased the gain and directivity of the antenna with minimal impact on its operating bandwidth (Figure 23a). Figure 23 compares the S11 and realized gain of RA2 and the resulting reflector-loaded antenna 2 (RLA2). The reflector size was set to 1.4 times that of RA2, adhering to the Yagi design principles. The radiating element was 60% of 0.45λ, and the reflector was 60% of 0.6λ, considering the minimum operating frequency.

Figure 23b reveals a significant increase in gain across the entire operating band, with a maximum increase of 3.15 dB at 2 GHz. This enhanced gain and directivity translated into stronger field strength inside the object to be imaged, leading to improved tumor sensing, image quality, and tumor location accuracy.

**Figure 24:**
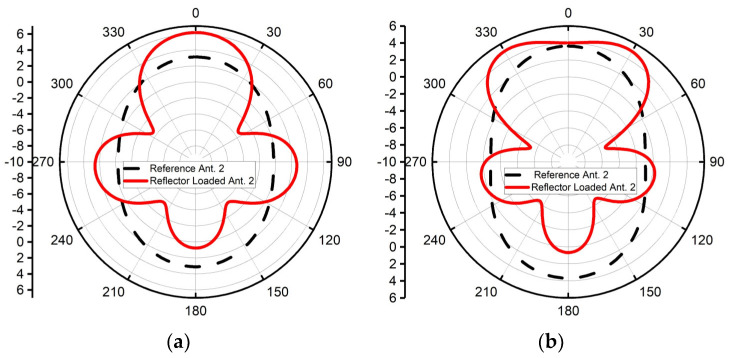

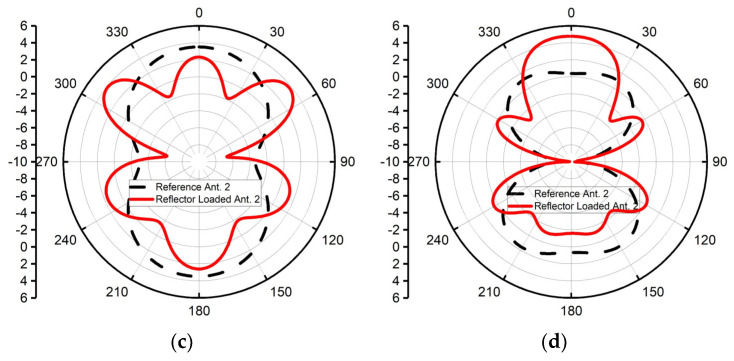
Radiation patterns of reference antenna 2 and reflector-loaded antenna 2 at (**a**) 2, (**b**) 2.5, (**c**) 3, and (**d**) 3.5 GHz.

**Figure 25:**
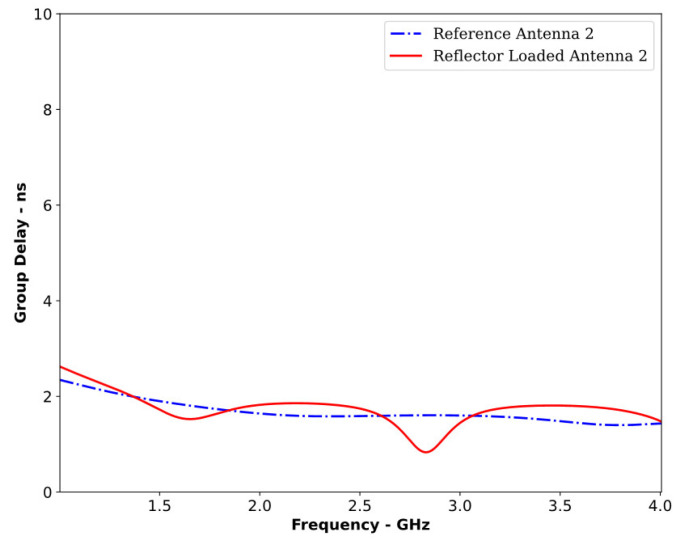
Group delay of RA2 and the proposed RLA2.

Furthermore, Figure 24 confirms that the RLA2 behaved as a directional antenna throughout its operating bandwidth. Figure 25 shows the group delay plot for both antennas, demonstrating that the RLA2 maintained comparable performance to that of the RA2.

In a face-to-face configuration when placed 250 mm apart, the correlation factor was 89.14% for RA2 and 84.71% for RLA2. These results provide evidence that the proposed reflector-loading technique successfully converted a non-directional antenna into a directional one with improved gain and directivity while preserving the time-domain performance and operating bandwidth, ultimately leading to better MMI results.

Therefore, with strong evidence and high confidence, it can be concluded that the reflector-loading technique effectively converts a wide range of non-directional microstrip patch antennas into their directional counterparts, leading to significant enhancements in MMI performance.

## 5. Comparison

While numerous studies regarding antennas for MMI applications exist, only a few have specifically addressed the impact of directivity and directional antennas on imaging results. Table 7 compares the proposed RLA with other directional antennas reported in the literature. It is evident that the RLA exhibits superior performance across various parameters.

Notably, the RLA achieves a maximum increase in signal strength of 14.26 dB compared to a non-directional antenna, significantly exceeding the 4 dB improvement reported in [31]. This remarkable improvement highlights the RLA’s potential for enhanced MMI performance.

Furthermore, unlike existing approaches, this work presents a general technique for enhancing gain and directivity that is applicable to a wide range of antennas. This innovative aspect significantly contributes to the novelty of the proposed approach.

Future work will involve utilizing advanced signal processing algorithms to reconstruct 2D images from backscattered signals for accurate tumor detection and localization.

## 6. Conclusions

Inspired by the high directivity of Yagi-Uda antennas, this work presented a novel and general technique for converting non-directional microstrip patch antennas into directional ones. This technique was applied to a UWB reference antenna that is known for its suitability in MMI applications. While the proposed size and location of the reflector were primarily based on Yagi-Uda design principles, slight deviations were observed, which were likely due to the specific geometry of the reference antenna. The resulting antenna, termed the RLA, validated the proposed reflector-loading technique and exhibited significantly improved directional radiation properties compared to those of the original antenna.

The subsequent analysis of this work investigated the application of the RLA in MMI by simulating the field distribution within a human breast model. The results revealed that the RLA achieved a remarkable increase of 14.26 dB in maximum field strength compared to the reference antenna, leading to improved signal penetration and potentially better imaging results.

Further investigation of the RLA’s time-domain properties in both the E-plane and H-plane confirmed its ability to preserve the input signal’s pulse shape with a maximum correlation factor of 94.86%, demonstrating exceptional time-domain performance.

These findings clearly establish the RLA as a promising candidate for MMI applications. To further validate the proposed technique, another reference antenna known for its MMI compatibility was loaded with a reflector. The resulting antenna showed significant enhancements in gain and directivity while maintaining favorable time-domain performance and operating bandwidth. This successful application further strengthens the validity and potential of the proposed reflector-loading technique for MMI applications.

## Figures and Tables

**Table 1:** Comparison of the performance parameters of the reference antenna and the proposed RLA.

Parameter	Maximum Value Associated with the Reference Antenna	Maximum Value Associated with the Proposed RLA (Simulated)	Maximum Value Associated with the Proposed RLA (Measured)
Operating Bandwidth	3.00 to 12 GHz	3.02 to 12 GHz	2.8 to 12 GHz
Realized Gain	4.3 dBi	7.3 dBi	7.8 dBi
Front-to-Back Ratio	7.5	14.6	12.9
Radiation Pattern	Non-directional throughout the operating band	Directional throughout the operating band	Directional throughout the operating band
Total Efficiency	83%	85%	80%

**Table 2:** Maximum E-field values inside the tumor with the antenna facing the breast model.

Frequency (GHz)	Maximum E-Field with the Reference Antenna (V/m)	Maximum E-Field with the Proposed RLA (V/m)	Increase in Field Intensity (dB)
3.5	0.36	1.87	14.26
4.6	0.36	1.56	12.73
5.0	0.25	0.96	11.68
6.5	0.26	1.16	12.98
8.0	0.32	1.26	11.90

**Table 3:** Maximum E-field values inside the tumor with antennas placed sideways with respect to the breast model.

Frequency (GHz)	Maximum E-Field with the Reference Antenna (V/m)	Maximum E-Field with the Proposed RLA (V/m)	Increase in Field Intensity (dB)
3.5	1.29	1.49	1.25
4.6	1.32	1.53	1.28
5.0	1.29	1.61	1.24
6.5	1.24	2.31	5.40
8.0	0.90	1.51	4.49

**Table 4:** Correlation factor for virtual probes placed in the E-plane (∅=90°).

Probe Position		Correlation Factor (%)
∅ = 90°	*θ* = −90°	95.67
∅ = 90°	*θ* = −45°	97.78
∅ = 90°	*θ* = 0°	98.79
∅ = 90°	*θ* = 45°	98.58
∅ = 90°	*θ* = 90°	80.00

**Table 5:** Correlation factor for virtual probes placed in the H-plane (∅=0°).

Probe Position		Correlation Factor (%)
∅ = 0°	*θ* = −90°	98.87
∅ = 0°	*θ* = −45°	94.40
∅ = 0°	*θ* = 0°	84.00
∅ = 0°	*θ* = 45°	94.21
∅ = 0°	*θ* = 90°	98.82

**Table 6:** Comparison of the performance of the reference antenna and the proposed RLA in various tests conducted to analyze their suitability for use in MMI systems.

Analyzed Parameter	Maximum Value Associated with the Reference Antenna	Maximum Value Associated with the Proposed RLA
Field strength inside the tumor	0.36 V/m	1.87 V/m
Time-domain pulse correlation factor	94.59%	94.86%
Difference between S21 in the absence and presence of a tumor	0.98 dB	2.15 dB

**Table 7:** Comparison of the proposed RLA with other antennas reported in the literature with a focus on enhancing antenna directivity for applications in MMI systems.

Reference	Antenna Size in mm (Electrical Size)	Operating Bandwidth	Peak Gain (dBi)	Correlation Factor	Max. Increase in the Penetrated Field Strength	Correlation Factor Calculated in Both the E-Plane and H-Plane	Generality of the Proposed Technique
[23]	110 × 110 × 48 (0.15λ × 0.15λ × 0.06λ)	0.43 to 1.85	N/A	N/A	11 dB	N/A	No
[24]	60 × 60 × 50 (0.2λ × 0.2λ×0.16λ)	1 to 6	N/A	80	N/A	N/A	No
[26]	40 × 40 (0.33λ × 0.33λ)	2.5 to 11	7.1	98	N/A	N/A	No
[31]	70 × 45 × 15 (0.29λ × 0.19λ×0.06λ)	1.25 to 2.4	~4	~90	5 dB	Yes	No
[37]	26.6 × 29 (0.34λ × 0.37λ)	3.8 to 10.1	6.8	91.6	N/A	N/A	No
[38]	45 × 37 (0.58λ × 0.48λ)	3.9 to 9.15	6.8	92	N/A	N/A	No
[39]	70 × 30 × 14 (0.26λ × 0.11λ×0.05λ)	1.12 to 2.5	5.192	80	N/A	N/A	No
[40]	25 × 25 × 10.5 (0.22λ × 0.22λ×0.09λ)	2.65 to 2.91	6.6	N/A	N/A	N/A	No
Proposed RLA	29.4 × 32.2 × 25 (0.22λ × 0.22λ×0.09λ)	3.02 to 12	7.3	94.86	14.26 dB	Yes	Yes, Proved

## Data Availability

Dataset available on request from the authors.

## References

[1] Islam M.T., Mahmud M.Z., Islam M.T., Kibria S., Samsuzzaman M. (2019). A low cost and portable microwave imaging system for breast tumor detection using UWB directional antenna array. Sci. Rep..

[2] Conceição R.C., Mohr J.J., O’Halloran M. (2016). An Introduction to Microwave Imaging for Breast Cancer Detection.

[3] Noghanian S., Sabouni A., Desell T., Ashtari A. (2014). Microwave Tomography: Global Optimization, Parallelization and Performance Evaluation.

[4] Fiser O., Helbig M., Sachs J., Ley S., Merunka I., Vrba J. (2018). Microwave non-invasive temperature monitoring using UWB radar for cancer treatment by hyperthermia. Prog. Electromagn. Res..

[5] Grzegorczyk T.M., Meaney P.M., Kaufman P.A., di Florio-Alexander R.M., Paulsen K.D. (2012). Fast 3-D tomographic microwave imaging for breast cancer detection. IEEE Trans. Med. Imaging.

[6] Tobon Vasquez J.A., Scapaticci R., Turvani G., Bellizzi G., Rodriguez-Duarte D.O., Joachimowicz N., Duchêne B., Tedeschi E., Casu M.R., Crocco L. (2020). A prototype microwave system for 3D brain stroke imaging. Sensors.

[7] Merunka I., Massa A., Vrba D., Fiser O., Salucci M., Vrba J. (2019). Microwave tomography system for methodical testing of human brain stroke detection approaches. Int. J. Antennas Propag..

[8] Rahama Y.A., Al Aryani O., Din U.A., Awar M.A., Zakaria A., Qaddoumi N. (2018). Novel microwave tomography system using a phased array antenna. IEEE Trans. Microw. Theory Tech..

[9] Dachena C., Fedeli A., Fanti A., Lodi M.B., Pastorino M., Randazzo A. (2021). Microwave imaging for the diagnosis of cervical diseases: A feasibility analysis. IEEE J. Electromagn. RF Microw. Med. Biol..

[10] Amin B., Shahzad A., Crocco L., Wang M., O’Halloran M., González-Suárez A., Elahi M.A. (2021). A feasibility study on microwave imaging of bone for osteoporosis monitoring. Med. Biol. Eng. Comput..

[11] Sachs J., Ley S., Just T., Chamaani S., Helbig M. (2018). Differential ultra-wideband microwave imaging: Principle application challenges. Sensors.

[12] Fear E.C., Li X., Hagness S.C., Stuchly M.A. (2002). Confocal microwave imaging for breast cancer detection: Localization of tumors in three dimensions. IEEE Trans. Biomed. Eng..

[13] Song H., Sasada S., Kadoya T., Okada M., Arihiro K., Xiao X., Kikkawa T. (2017). Detectability of breast tumor by a hand-held impulse radar detector: Performance evaluation and pilot clinical study. Sci. Rep..

[14] Baran A., Kurrant D.J., Zakaria A., Fear E.C., LoVetri J. (2014). Breast imaging using microwave tomography with radar-based tissue-regions estimation. Prog. Electromagn. Res..

[15] Sabouni A., Flores-Tapia D., Noghanian S., Thomas G., Pistorius S. Hybrid microwave tomography technique for breast cancer imaging. Proceedings of the 2006 International Conference of the IEEE Engineering in Medicine and Biology Society.

[16] Bourqui J., Okoniewski M., Fear E.C. (2010). Balanced antipodal Vivaldi antenna with dielectric director for near-field microwave imaging. IEEE Trans. Antennas Propag..

[17] Li X., Hagness S.C., Choi M.K., Weide D.W.V.D. (2003). Numerical and experimental investigation of an ultrawideband ridged pyramidal horn antenna with curved launching plane for pulse radiation. IEEE Antennas Wirel. Propag. Lett..

[18] Di Clemente F.S., Helbig M., Sachs J., Schwarz U., Stephan R., Hein M.A. Permittivity-matched compact ceramic ultra-wideband horn antennas for biomedical diagnostics. Proceedings of the 5th European Conference on Antennas and Propagation (EUCAP).

[19] Jafari H.M., Deen M.J., Hranilovic S., Nikolova N.K. (2007). A study of ultrawideband antennas for near-field imaging. IEEE Trans. Antennas Propag..

[20] Alam A.H.M.Z., Islam M.R., Khan S. Design and analysis of UWB rectangular patch antenna. Proceedings of the 2007 Asia-Pacific Conference on Applied Electromagnetics.

[21] Yurduseven O., Smith D., Elsdon M. (2013). Printed slot loaded bow-tie antenna with super wideband radiation characteristics for imaging applications. IEEE Trans. Antennas Propag..

[22] Kanj H., Popovi’c M. (2005). Miniaturized microstrip-fed ‘dark eyes’ antenna for near-field microwave sensing. IEEE Antennas Wirel. Propag. Lett..

[23] Mousavi S.M.H., Rezaeieh S.A., Darvazehban A., Mohammed B., Janani A.S., Abbosh A.M. (2022). Tapered Graded Index Lens Antenna with Enhanced Penetration for Near-Field Torso Imaging. IEEE Trans. Antennas Propag..

[24] Fiser O., Hruby V., Vrba J., Drizdal T., Tesarik J., Vrba J., Vrba D. (2022). UWB bowtie antenna for medical microwave imaging applications. IEEE Trans. Antennas Propag..

[25] Sediq H.T. (2023). Tumor detection concepts using eagle-shaped UWB antenna signals for medical purposes. Sens. Actuators A Phys..

[26] Samsuzzaman M., Islam M.T., Islam M.T., Shovon A.A., Faruque R.I., Misran N. (2019). A 16-modified antipodal Vivaldi antenna array for microwave-based breast tumor imaging applications. Microw. Opt. Technol. Lett..

[27] Li Q., Liu Z., Zhao Y., Zhao R., Fan M., Zhang G., Yu M., Han P. (2023). A Portable Microwave Intracranial Hemorrhage Imaging System Based on PSO-MCKD-CEEMDAN Method. IEEE Trans. Microw. Theory Tech..

[28] Amin B., Shahzad A., O’halloran M., Mcdermott B., Elahi A. (2022). Experimental Validation of Microwave Imaging Prototype and DBIM-IMATCS Algorithm for Bone Health Monitoring. IEEE Access.

[29] Guo L., Alqadami A.S.M., Abbosh A. (2023). Stroke Diagnosis Using Microwave Techniques: Review of Systems and Algorithms. IEEE J. Electromagn. RF Microw. Med. Biol..

[30] Islam M.T., Samsuzzaman M., Rahman M.N., Islam M.T. (2018). A compact slotted patch antenna for breast tumor detection. Microw. Opt. Technol. Lett..

[31] Mobashsher A.T., Abbosh A.M. (2016). Performance of directional and omnidirectional antennas in wideband head imaging. IEEE Antennas Wirel. Propag. Lett..

[32] Martínez-Lozano A., Blanco-Angulo C., García-Martínez H., Gutiérrez-Mazón R., Torregrosa-Penalva G., Ávila-Navarro E., Sabater-Navarro J.M. (2021). UWB-Printed Rectangular-Based Monopole Antenna for Biological Tissue Analysis. Electronics.

[33] Balanis C.A. (2016). Antenna Theory: Analysis and Design.

[34] Mehdipour A., Mohammadpour-Aghdam K., Faraji-Dana R., Kashani-Khatib M.R. (2008). A novel coplanar waveguide-fed slot antenna for ultrawideband applications. IEEE Trans. Antennas Propag..

[35] Islam M.M., Islam M.T., Faruque M.R.I., Samsuzzaman M., Misran N., Arshad H. (2015). Microwave imaging sensor using compact metamaterial UWB antenna with a high correlation factor. Materials.

[36] Alqahtani A., Islam M.T., Talukder M.S., Samsuzzaman M., Bakouri M., Mansouri S., Almoneef T., Dokos S., Alharbi Y. (2022). Slotted monopole patch antenna for microwave-based head imaging applications. Sensors.

[37] Zerrad F.E., Taouzari M., Makroum E.M., Aoufi J.E., Qanadli S.D., Karaaslan M., Al-Gburi A.J.A., Zakaria Z. (2023). Microwave Imaging Approach for Breast Cancer Detection Using a Tapered Slot Antenna Loaded with Parasitic Components. Materials.

[38] Mahmud M.Z., Islam M.T., Samsuzzaman M., Kibria S., Misran N. (2017). Design and parametric investigation of directional antenna for microwave imaging application. IET Microw. Antennas Propag..

[39] Islam M.S., Islam M.T., Hoque A., Islam M.T., Amin N., Chowdhury M.E. (2021). A portable electromagnetic head imaging system using metamaterial loaded compact directional 3D antenna. IEEE Access.

[40] Rokunuzzaman M., Samsuzzaman M., Islam M.T. (2016). Unidirectional wideband 3-D antenna for human head-imaging application. IEEE Antennas Wirel. Propag. Lett..

